# The Book World of Medicine and Science

**Published:** 1907-04-06

**Authors:** 


### The Book World of Medicine and Science.

Climatology and Balneotherapy. By Sir Herman Weber and F. Parkes Weber, M.D. (Smith, Elder and Co., 15 Waterloo Place, S.W. 15s. net.)

This, the third edition of Dr. Weber's "Mineral Waters and Health Resorts of Europe," has been much, and usefully, enlarged in respect to medical climatology. The first part now deals with health resorts, giving at the same time an excellent chapter on ocean climates and sea voyages. This chapter, indeed, calls for more than mere mention. It deals exhaustively with the cases which are suitable for sea voyages, and with the contra-indications. Among the latter the authors include epilepsy, which is, however, sometimes ameliorated by a sea voyage. We are somewhat surprised, too, to see that so little is said about the Indian route, which is nowadays comparatively cheap, and certainly one of the most interesting sea trips that the convalescent can take. The chapters on specific health resorts are well written, and contain much useful information, notes, that is, which will be informative not only to the practitioner but to the patient as well.

The second part, dealing with spas and balneotherapy in general, is more technical, and includes full descriptions of the many spas to which the practitioner may send his patient. Specially worthy of note are the short paragraphs dealing with precautions to be taken. The third part, perhaps the most interesting and useful in the book, is devoted to "Indications for Treatment," the chapter on tuberculosis being remarkable for its lucid style and its many practical hints which tempt one to quote beyond the limitations of available space. In addition to these useful features the book possesses a very exhaustive bibliography, and—what is of much more importance to the busy practitioner—an index which is one of the best arranged that we have yet met with in a scientific work. As a handbook on the subjects with which it deals, the volume is in every way to be commended, and it will well repay perusal. It is one of those books that ought to figure prominently on the shelves of the general practitioner.

Food and Digestion in Health and Disease. By M. A. Dutch, M.D. (London: Henry Kimpton, 1906. Pp. 244. Price 2s. net.)

In addition to the above subjects, there are discussed in this book the nature of matter, the phenomena of life, and the source, properties, and influence of water. It is intended apparently for the instruction of nurses and of lay people who desire to know something of food and digestion. Chemistry, physiology, and anatomy are all touched on in the opening chapters. The various food materials and their nutritive values are described, and special dietaries for different ages are recommended. The author has brought together from many sources a large number of facts bearing on the subjects of food and feeding, and has arranged them in a very readable form. When he treats of food in disease he is on more debatable ground, and is dealing with a subject which is so purely medical that it is not suitable for the general reader. The numerous tables, showing the chemical analyses of foods and their nutritive values, are quite out of place in a book of this nature. We do not notice much that is new in these pages, but nurses may find some information which is useful to them.

Healthy Homes. By A. G. Magian, M.D., B.Ch., F.R.G.S. (John Heywood, Limited, Manchester, and Lamb's Conduit Street, London, E.C. Price 1d.)

Dr. Magian's booklet is a good pennyworth. He avoids technical phrases and elaborate "reasons why," and his advice on the choice of a house, having due regard to the soil on which it is built, its aspect, surroundings, and neighbouring open spaces is clear, practical, and reasonable. When he touches upon furnishing, Dr. Magian ventures on ground where most men, whether doctors or not, fear to tread; but even the most conservative housekeeper may take a hint or two from his pages without being carried off to those chilly heights of sanitary discomfort which many writers insist on. Dr. Magian says that his pamphlet "aims at getting at the working and artisan classes, who *may* spend a penny on such a book, but at first, at all events, cannot be induced to spend more." But even those who might be inclined to greater lavishness will find this little work simple, clear, and compact. It is well suited to the intelligence of the plain man or woman who has no pretensions to special knowledge, but who wants none the less to have a healthy home.

